# Nck adapter proteins promote podosome biogenesis facilitating extracellular matrix degradation and cancer invasion

**DOI:** 10.1002/cam4.2640

**Published:** 2019-10-22

**Authors:** Sankar P. Chaki, Rola Barhoumi, Gonzalo M. Rivera

**Affiliations:** ^1^ Department of Veterinary Pathobiology Texas A&M University College Station TX USA; ^2^ Department of Veterinary Integrative Biosciences Texas A&M University College Station TX USA

**Keywords:** cancer, c‐Src, Dok1, extracellular matrix, Nck, podosome

## Abstract

**Background:**

Podosomes are membrane‐bound adhesive structures formed by actin remodeling. They are capable of extracellular matrix (ECM) degradation, which is a prerequisite for cancer cell invasion and metastasis. The signaling mechanism of podosome formation is still unknown in cancer. We previously reported that Nck adaptors regulate directional cell migration and endothelial lumen formation by actin remodeling, while deficiency of Nck reduces cancer metastasis. This study evaluated the role of Nck adaptors in podosome biogenesis and cancer invasion.

**Methods:**

This study was conducted in vitro using both healthy cells (Human Umbilical Vein Endothelial Cell, 3T3 fibroblasts) and cancer cells (prostate cancer cell line; PC3, breast cancer cell line; MDA‐MB‐231). Confocal and TIRF imaging of cells expressing Green Fluorescence Protein (GFP)  mutant under altered levels of Nck or downstream of kinase 1 (Dok1) was used to evaluate the podosome formation and fluorescent gelatin matrix degradation. Levels of Nck in human breast carcinoma tissue sections were detected by immune histochemistry using Nck polyclonal antibody. Biochemical interaction of Nck/Dok1 was detected in podosome forming cells using immune precipitation and far‐western blotting.

**Results:**

This study demonstrates that ectopic expression of Nck1 and Nck2 can induce the endothelial podosome formation in vitro. Nck silencing by short‐hairpin RNA blocked podosome biogenesis and ECM degradation in cSrc‐Y530F transformed endothelial cells in this study. Immunohistochemical analysis revealed the Nck overexpression in human breast carcinoma tissue sections. Immunoprecipitation and far‐western blotting revealed the biochemical interaction of Nck/p62Dok in podosome forming cells.

**Conclusions:**

Nck adaptors in interaction with Dok1 induce podosome biogenesis and ECM degradation facilitating cancer cell invasion, and therefore a bona fide target of cancer therapy.

## Graphical abstract

This study evaluated the role of Nck adaptors in podosome biogenesis, capable of extracellular matrix (ECM) degradation, required in cancer metastasis. Ectopic expression of Nck1 and Nck2 induces endothelial podosome formation and ECM degradation whereas Nck silencing blocks the process. Immunohistochemical analysis revealed the Nck overexpression in human breast carcinoma tissue sections. Immunoprecipitation and far‐western blotting revealed the biochemical interaction of Nck/p62Dok in podosome forming cells. In conclusion, Nck adaptors in interaction with downstream of kinase 1 induce the podosome formation and podosome‐mediated ECM degradation facilitating cancer cell invasion, and therefore, a bona fide target of cancer therapy.

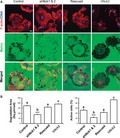

## 1 INTRODUCTION

Actin‐rich subcellular structures, podosomes and invadopodia, are capable of degrading extracellular matrix (ECM) facilitating invasive cell migration.^1^ ECM remodeling and cell migration are prerequisites for new blood vessel formation, cardiovascular disease, and cancer metastasis. Podosomal structures are found in macrophages, endothelial cells, osteoclasts, dendritic cells, and smooth muscle cells.^1^, ^2^ Highly dynamic nature of podosomes generates a diffuse pattern of matrix degradation. Invadopodia in cancer cells,are more stable (hours) and capable of focalized matrix degradation. Endothelial cells barely form podosomes in vitro, but their formation can be stimulated by VEGF, TGF‐β, and PMA (phorbol‐12‐myristate‐13‐acetate). Large rosette‐like podosomal structures are formed in Src‐transformed fibroblasts,^3^ osteoclasts,^4^ macrophages,^5^ endothelial cells,^6^, ^7^, ^8^ and invasive cancer cells. Podosome forms in the native endothelium of arterial vessels when exposed to TGFβ.^8^ Furthermore, formation of podosome during blood vessel formation,^9^ as well as vascular branching,^10^ suggest a critical role for these structures in vascular morphogenesis. Also, podosome‐mediated vascular smooth muscle cells migration occurs during the formation of the atherosclerotic lesion.^11^ Thus understanding the molecular mechanisms of podosome biogenesis is critical for the development of disease‐modifying therapies.

Signaling pathways regulating podosome formation and podosome‐mediated ECM remodeling are not well‐characterized. Cancer metastasis is a condition which requires ECM degradation and cellular invasion. Earlier, we and others have shown the involvement of Nck actin regulator in tumorigenesis and metastasis.^12^, ^13^, ^14^ This study emphasizes the role of Nck in podosome biogenesis and podosome‐mediated ECM degradation, which is necessary for cancer cell invasion. Nck initiates actin polymerization in invadopodia through the activation of N‐WASp/Arp 2/3 pathway in cancer cells.^15^, ^16^, ^17^ Previous studies show the involvement of phosphorylated Tks5^15^ and cortactin^18^ in the recruitment of Nck adaptors to actin‐based invadopodia. Interestingly, Nck1, which is involved in podosome‐independent matrix invasion,^19^ identified as a specific marker of invadopodia but not podosomes.^20^

In contrast to the notion that Nck adaptors are critical regulators of invadopodia, their role in podosome biogenesis remains mostly undetermined. The scaffolding protein downstream of kinase 1 (Dok1) has been implicated in the regulation of cell proliferation and cytoskeletal rearrangements.^21^ Dok1 phosphorylation triggers the recruitment of Nck and the formation of filopodia.^22^ Inhibition of B16F10 mouse melanoma cell migration by dominant‐negative mutants of Dok1^23^ suggests an essential role of this scaffolding protein in cytoskeletal pathways controlling cells motility and invasion. Thus, it is vital to study Dok1/Nck complex formation in podosome biogenesis.

## 2 MATERIALS AND METHODS

### 2.1 Reagents

Antibodies used were mouse anti‐Nck (BD Biosciences), mouse anti‐GAPDH (Invitrogen), mouse anti‐β actin (Sigma), and goat anti‐mouse/ anti‐rabbit IgG‐HRP (Santa Cruz Biotechnology). EGM2 cell culture medium, Trypsin Neutralizing Solution, and Hank's Balanced Salt Solution were purchased from Lonza. Other reagents used were Fibronectin (Calbiochem/Invitrogen), recombinant human VEGF 165 (R&D Systems), PMA (Sigma Aldrich), Texas Red phalloidin (Invitrogen), lipofectamine 2000 (Invitrogen), DMEM (HyClone), fetal bovine serum (BenchMark), Alexa Fluor 488 protein labeling kit (A10235 Invitrogen), DAB (3,39‐diaminobenzidine) (DakoCytomation), and Transwell inserts (20 mm diameter, 8.0 μm pore size polycarbonate membrane, Corning, NY).

### 2.2 Cell culture

HUVEC were cultured in EGM2 complete media with 2% FBS and 1% penicillin/streptomycin, NIH 3T3 fibroblasts were cultured in DMEM with 10% FCS and 1% penicillin/streptomycin, and HEK293T cells were cultured in DMEM with 10% FBS and 1% penicillin/streptomycin. Cells were incubated at 37°C with 5% CO_2_. Every alternate day, old media was replaced with fresh media. Starvation media contained 1/10th of the serum of complete media.

### 2.3 Plasmids and viral transduction

P‐Super (puro/hygro) retroviral vector for stable gene knockdown and pMSCV (puro/hygro) retroviral vector for stable protein expression of nontagged or fluorescently tagged proteins were used in this study. HEK293T cells, pre‐treated with polybrene, were co‐transfected by calcium phosphate precipitation with plasmids carrying shRNA nucleotide sequence or cDNA sequence, pHCMV‐G, and pMD.gag.pol plasmids. Virus medium was collected after 24 hours of transfection till 48 hours and stored in aliquots at −80°C for future use. Viral transduction of cells was performed as per the method described earlier.^24^

### 2.4 Western immunoblotting

Western blotting was performed as per the method described previously.^25^ Briefly, cells were harvested in ice‐cold kinase lysis buffer containing protease and phosphatase inhibitors. Equal amounts of protein (3‐5 µg) as measured in the cell supernatant were resolved in 12% SDS‐PAGE. Proteins transferred into nitrocellulose membranes were blocked in 0.5% nonfat dry milk (1‐2 hours at room temperature), probed with primary antibody (4°C overnight) followed by washing and incubation with HRP‐tagged secondary antibody (1 hour at room temperature). Washed membranes were incubated in enhanced chemiluminescence substrate to capture images using a Luminescent Image Analyzer. Image analysis was performed using EMBL ImageJ.

### 2.5 Imaging

Images of fixed samples were collected using a Zeiss LSM 510/Zeiss LSM 780 confocal microscope and Zeiss TIRF 3 microscope with Plan‐Neofluar 40×/1.3 oil objective and Apochromat 100×/1.46 oil objective. Time‐lapse series of podosome adhesion structures were captured every 15 seconds up to 90 minutes using a Zeiss TIRF 3 microscope under Plan‐Apochromat 100×/1.46 oil objective and equipped with EMCCD camera.

### 2.6 RNA isolation and reverse transcriptase (RT)‐PCR

Confluent cells cultured in 10 cm plate for 3 days were washed with DPBS and RNA were isolated using a commercially available kit (RNeasy kit, QIAGEN). Intron‐spanning MT1MMP primer was designed from sequence obtained from NCBI (NM_004995.2) for RT‐PCR; sense primer 5′‐GAGCTCAGGGCAGTGGATAG‐3′, antisense primer 5′‐GGTAGCCCGGTTCTACCTTC‐3′ (product size; cDNA 172 bp and genomic DNA 513 bp). GAPDH housekeeping gene primers were designed using sequence from NCBI (NM_002046.3); sense primer 5′‐AGAAGGTGGTGAAGCAGGCGTCGGAGG‐3′, antisense primer 5′‐AAGGTGGAGGAGTGGGTGTCGCTGTTGAAG‐3′ (product size; 109 bp).

RT‐PCR reactions were carried out as per the method described previously^26^ using Superscript III One‐Step RT‐PCR System and Platinum Taq DNA polymerase (Invitrogen), 40 pmol of each primer, and 100 ng of mRNA. PCR was carried out with cycle set up: One cycle of 50°C for 30 minutes, one cycle of 94°C for 2 minutes, 30 cycles of 94°C for 15 seconds, 58°C for 30 seconds, and 68°C for 1 minute with a final extension at 68°C for 5 minutes. RT‐PCR products were resolved and visualized on 2.0% agarose gels.

### 2.7 Fluorescent gelatin matrix degradation

Gelatin labeling using a kit from Invitrogen and matrix degradation assay were performed as per the protocol described previously.^27^ After labeling, one part of Alexa Fluor 488 labeled gelatin was diluted with eight parts of 0.2% unlabeled gelatin in DPBS at 37°C to coat poly‐l‐lysine (50 mg/mL) coated glass coverslip and quenched with sodium borohydride (5 mg/mL). Src‐transformed HUVEC (5 × 10^4^ cells) from different experimental groups were incubated overnight in 1 mL EGM‐2 complete media on washed gelatin‐coated coverslips in 24‐well culture plates. Following fixation in 4% paraformaldehyde, permeabilization in 0.2% TritonX100, and blocking in 2% BSA, cells were stained with Texas Red phalloidin (1:200) for 1 hour at room temperature. Images were collected using an inverted confocal microscope under 40×/1.3 NA oil immersion objective lens.

### 2.8 Transwell cell invasion

Cell invasion assay was performed using a 24 wells Corning Transwell cell culture plate as previously described^28^ with slight modifications. Briefly, the outer surface of the insert was coated with 50 µL of fibronectin (10 μg/mL) for 1 hour at room temperature, and the excess liquid was gently aspirated. The inner surface was coated with 50 µL of Matrigel^®^ (125 μg/mL) at room temperature for an hour. Human Umbilical Vein Endothelial Cell (HUVEC) were serum‐starved for 6 hours and seeded (4 × 10^4^ cells) on the coated insert in the upper chamber with starvation media. The bottom chamber of each well contained 50 ng/mL of VEGF in starvation medium. Cells were incubated overnight at 37°C with 5% CO_2_, and nonmigrating cells were removed from the inner side of the insert using a Q‐Tip cotton swab. Paraformaldehyde (3.8%) fixed inserts were stained (0.1% Crystal violet in PBS, 2% ethanol) for 5 minutes at room temperature. Images were collected using a 20× bright field objective and analyzed using EMBL ImageJ.

### 2.9 Wound healing assay

Wound healing assay was performed as per the method described previously.^29^, ^30^ Briefly, wound created (using a 200 μL sterile tip) on a confluent monolayer of serum‐starved HUVEC on fibronectin‐coated surfaces was allowed to repair in the presence of starvation medium supplemented with VEGF (50 ng/mL). Following overnight incubation, images were collected under brightfield objective to measure wound repair. Images were processed, and data were analyzed using EMBL.

### 2.10 Analysis of podosome dynamics

HUVECs expressing Nck2/YFP‐actin/mCherry or Nck1/YFP‐NCK2/mCherry or Dok1/YFP‐LifeAct/mCherry were seeded at a density of 10 × 10^4^ cells on fibronectin‐coated glass‐bottom MatTek dishes. Following 3‐5 hours of incubation, TIRF images of live cells were collected every 15 seconds up to 90 minutes. Six to 10 cells were analyzed per experimental condition using EMBL ImageJ or Fiji.

### 2.11 Immunohistochemical analysis of Nck expression in human breast carcinoma tissue

Immunohistochemical localization of Nck was performed in human breast carcinoma tissue array (BioChain Institute, Inc, Cat No. Z7020005, Lot No. B110013) as per the protocol described earlier.^31^ In brief, breast carcinoma microarray tissue sections (70 cases in duplicate) were deparaffinized and subjected to antigen unmasking in citrate buffer (pH 6) at 97°C water bath for 20 minutes. Slides were incubated with Nck primary antibody (1:200) for 1 hour at room temperature followed by 20 minutes incubation of biotinylated secondary antibody. The color was developed using streptavidin‐conjugated horseradish peroxidase and DAB (3,39‐diaminobenzidine) (DakoCytomation). Images were collected using a brightfield microscope and processed using EMBL ImageJ.

### 2.12 Statistics

Each experiment was repeated two to three times independently. Statistical analyses were performed using analysis of variance followed by suitable post HOC tests (eg, Tukey's test) in Minitab 16. Bar diagrams correspond to mean ± SD. The bold central lines of box plots indicate the median values, whereas the top and bottom lines indicate the third and first quartiles, respectively. The whiskers extend up to 1.5 times the interquartile range. Data were considered significant at *P* ≤ .05.

## 3 RESULTS

### 3.1 Nck localizes to actin‐rich podosomes of endothelial cells

**Figure 1:**
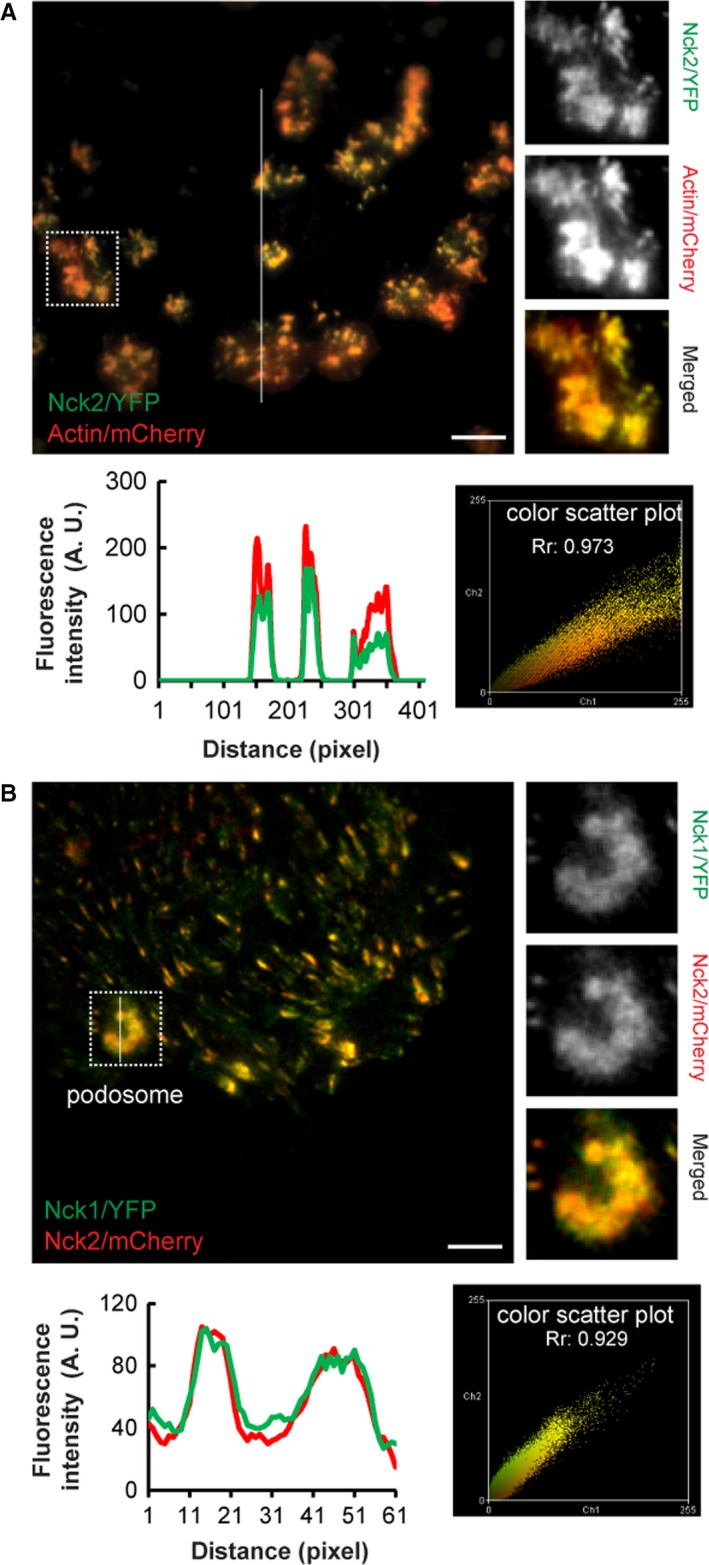
Nck localizes to actin‐rich podosome rosettes of Src‐transformed endothelial cells. A, Representative TIRF images of cell co‐expressing hNck2‐YFP (green) and actin‐mCherry (red) fusion proteins show colocalization of Nck with actin‐rich podosomes (yellow). The right panel of images corresponds to the square selection in the left image. Line intensity scan (bottom left) corresponding to the white line in the merged image as well as scatter plot (bottom right) corresponding to the whole‐cell image (top left) illustrating the relative colocalization of Nck2 and F‐actin in podosome rosettes (Pearson's correlation coefficient *R*r = 0.973). B, Representative TIRF images of cell co‐expressing hNck1‐YFP (green) and hNck2‐mCherry (red) fusion proteins show colocalization of Nck1 and Nck2 with podosomal rosettes (yellow). The right panel of images corresponds to the square selection in the left image. Line scan (bottom left) corresponding to the white line and scatter plot (bottom right) corresponding to the whole‐cell image illustrating the relative colocalization of Nck1 and Nck2 in podosome rosettes (Pearson's correlation coefficient *R*r = 0.929). Scale bars: 10 µm

Actin core in the center of podosomes is surrounded by a ring of adhesion and scaffold proteins like integrin, paxillin, and vinculin.^32^ Podosome puncta/ rosettes were characterized by colocalization of F‐actin and cortactin (Figure S1) in cSrc‐transformed endothelial cells, which are described as components of podosomes.^33^ We previously reported that Nck colocalizes with paxillin, a component of podosomes.^30^ We started analyzing Nck localization in podosomes. Constitutively active Src‐transformed endothelial cells can assemble podosomes^7^ and considered as a suitable model to study podosomes.^34^ TIRF imaging of Src‐transformed endothelial cells, co‐expressing Nck2‐EYFP, and actin‐mCherry showed a high level of localization of Nck2 to actin‐rich podosome rosettes. Calculated Manders correlation coefficient (*R*r) of 0.847 ± 0.0854 (mean ± SD; n = 55 podosomal rosettes), fluorescence intensity and scatter plot indicates significant colocalization of Nck in podosomal rosettes (Figure 1A). When normal endothelial cells were co‐expressed with Nck1‐EYFP and Nck2‐mCherry, strikingly high levels of colocalization of Nck1 and Nck2 in podosomal rosettes were also observed. Calculated Manders correlation coefficient (*R*r) of 0.881 ± 0.0868 (mean ± SD; n = seven cells covering 1408‐time frames of 15 seconds) and color scatter plot indicates significant colocalization of Nck isoforms with podosomal structures (Figure 1B).

### 3.2 Nck overexpression induces podosome biogenesis

To ascertain whether Nck can trigger podosome formation, we examined podosome dynamics in vitro in endothelial cells expressing Nck1‐YFP and Nck2‐mCherry. Endothelial cells co‐expressing Nck1‐YFP and Nck2‐mCherry showed podosome biogenesis and accumulation/colocalization of both proteins in podosomes (Figure 1B). Time‐lapse live‐cell TIRF imaging revealed highly dynamic podosomal rosettes formation that involved de novo podosome biogenesis as well as podosome formation by fission and fusion processes (Figure S2 and Movie S1). Thus, Nck expression can induce the podosome formation and be used as a suitable model of studying podosomes in vitro.

### 3.3 Depletion of endogenous Nck disrupts cSrc Y530F‐induced podosomes formation

**Figure 2:**
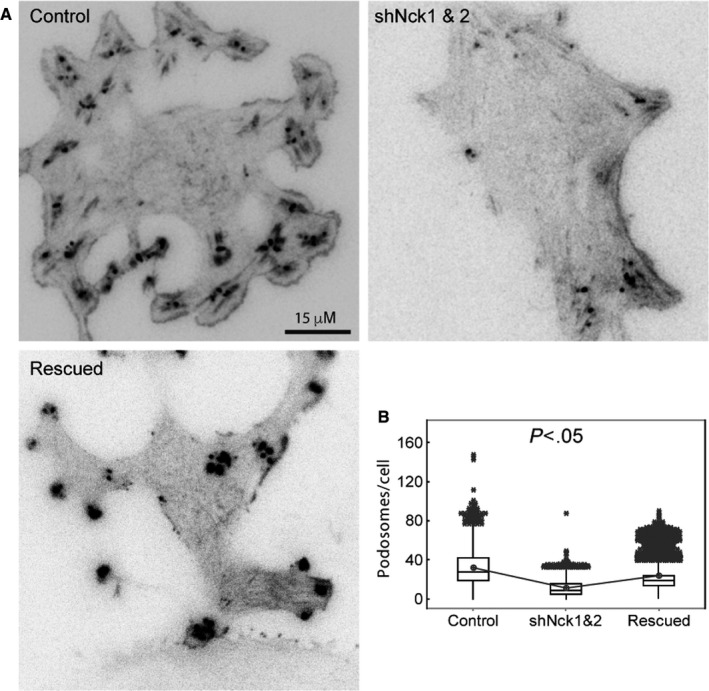
Silencing of Nck disrupted Src‐induced podosome formation in endothelial cells. A, Representative TIRF images of cSrc‐Y530F transformed endothelial cells shows very few podosomes in Nck silenced cells compared with control and rescued cells. B, Quantitative analysis indicates a significant decrease in podosome number in Nck‐depleted (shNck 1 and 2) cells compared with control and rescued cells. *P* < .05. The top and bottom lines of the box plot indicate the third and first quartiles, respectively, while the bold central lines indicate the median values.

Unlike macrophages, endothelial cells poorly form podosomes constitutively in vitro. Src transformation, PMA stimulation, VEGF activation, and TGFB treatment are commonly used to study the podosome formation and dynamics in different cell types.^7^, ^10^, ^35^, ^36^, ^37^ We first analyzed the podosome induction in endothelial cells by cSrc Y530F transformation and PMA stimulation. Podosomes were identified as actin puncta of ∼0.5 µm.^38^ In this study, 98 ± 1.5% (mean ± SD, n = three independent experiments) of cSrc Y530F‐transformed human umbilical vein endothelial cells, plated on a fibronectin‐coated substrate, formed podosomes puncta/ rosettes uniformly. In contrast, 50 ± 5.5% of cells showed diffuse podosomes formation after 4 hours of PMA stimulation (n = 10 cells per time points), (Figure S3). This suggests Src transformation is a better model of uniform podosome formation in vitro. In this study, we silenced Nck in cSrc‐transformed endothelial cells by retroviral expression of shNck1 and shNck2. Silencing of Nck causes drastic fall in podosome formation in cSrc‐transformed cells (Figure 2A). The number of podosomes per cell significantly (*P* < .05) dropped upon Nck depletion (Figure 2B). The cells expressing shNck1 and shNck2 had a mean of only 8 ± 1.7 (n = 96 cells) podosomes/cell. Notably, the effect of shNck1 and shNck2 on podosome formation was specific as it could be reversed by exogenous expression of mouse Nck1 and Nck2 insensitive to human shNck1 or human shNck2 (Figure 2A, bottom panel). Thus Nck is required in Src‐induced podosome formation.

### 3.4 Nck adaptors play an essential role in podosome‐mediated matrix remodeling

**Figure 3:**
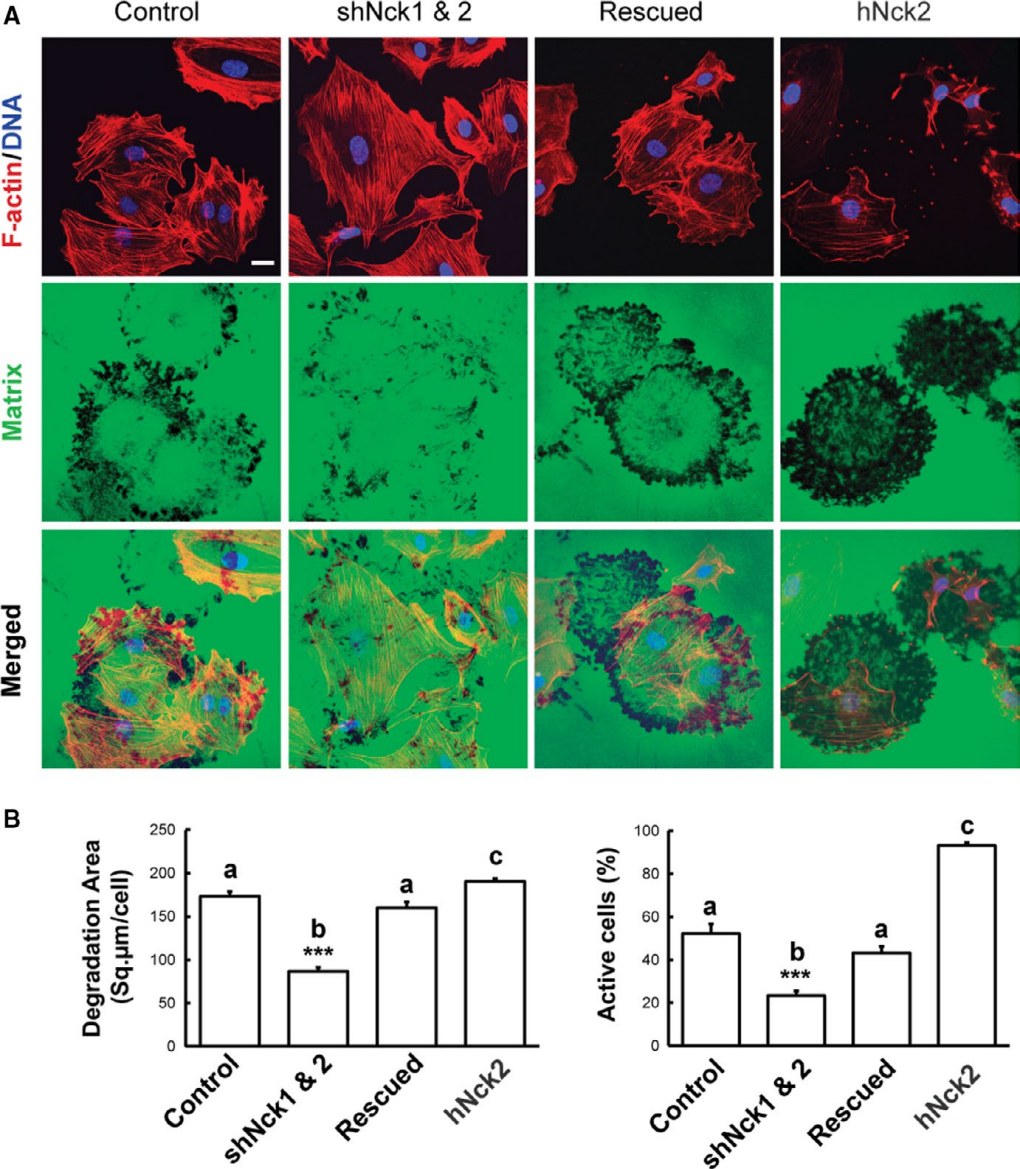
Nck adaptors play a critical role in podosome‐mediated extracellular matrix remodeling by endothelial cells. A, Representative confocal images are showing the degradation of a fluorescent matrix (Alexa Fluor 488‐conjugated gelatin) by podosomes induced by Src expression in HUVEC. Control, Nck‐rescued, and Nck‐overexpressing cells (hNck2) exhibit increased matrix degradation relative to the cells with simultaneous knockdown of Nck1 and Nck2 (shNck 1 and 2). Scale bar represents 20 µm. B, Quantitative image analysis showing decreased (*P* < .001) matrix degradation/cell (left panel) and several actively degrading cells (right panel) in Nck1/Nck2 knockdown (shNck 1 and 2) cells vs control, rescued, and Nck‐overexpressing cells. Bars represent mean ± SEM (n = 3 independent experiments). ***P < .001

Although podosomes may play an important role in matrix remodeling during cancer metastasis, the mechanism of podosomes assembly is still unknown.^39^ Microvascular endothelial cells can form podosomes without specific stimulation.^6^ Nck1 has been implicated in invadopodia formation and matrix degradation in various tumor cells, including breast carcinoma and melanoma cells.^15^, ^16^, ^40^ Endothelial cells form podosomes, in response to various stimuli in vitro^7^, ^41^, ^42^ and in vivo.^8^ Importantly, Src is involved in endothelial podosome formation.^7^ In this study, we first performed TIRF imaging of Src‐transformed HUVEC expressing fluorescence‐tagged Nck2. A high level of localization of Nck2 to actin‐rich podosome rosettes (Figure 1) prompted us to investigate the role of Nck1 and Nck2 in ECM degradation associated with Src‐induced podosome formation in HUVEC. Using a previously described assay of fluorescent matrix (gelatin) degradation,^27^ we observed that knockdown of Nck1, Nck2, or both induced a significant (*P* < .05) decrease in matrix degradation and the phenotype was rescued entirely by the expression of Nck2 (Figure 3A,B) and to a lesser extent by Nck1 (data not shown). Furthermore, overexpression of Nck induced significantly high (*P* < .05) level of matrix degradation than control. Also, a high level of overlapping between F‐actin and matrix degradation area was observed (Figure 3A). Due to cellular dynamics, some matrix degradation areas were devoid of F‐actin colocalization. Altogether, Nck adaptors play an essential role in podosome‐mediated matrix remodeling and cellular invasion.

### 3.5 Podosome‐mediated ECM remodeling by Nck facilitates chemotactic migration and invasion of endothelial cells

**Figure 4:**
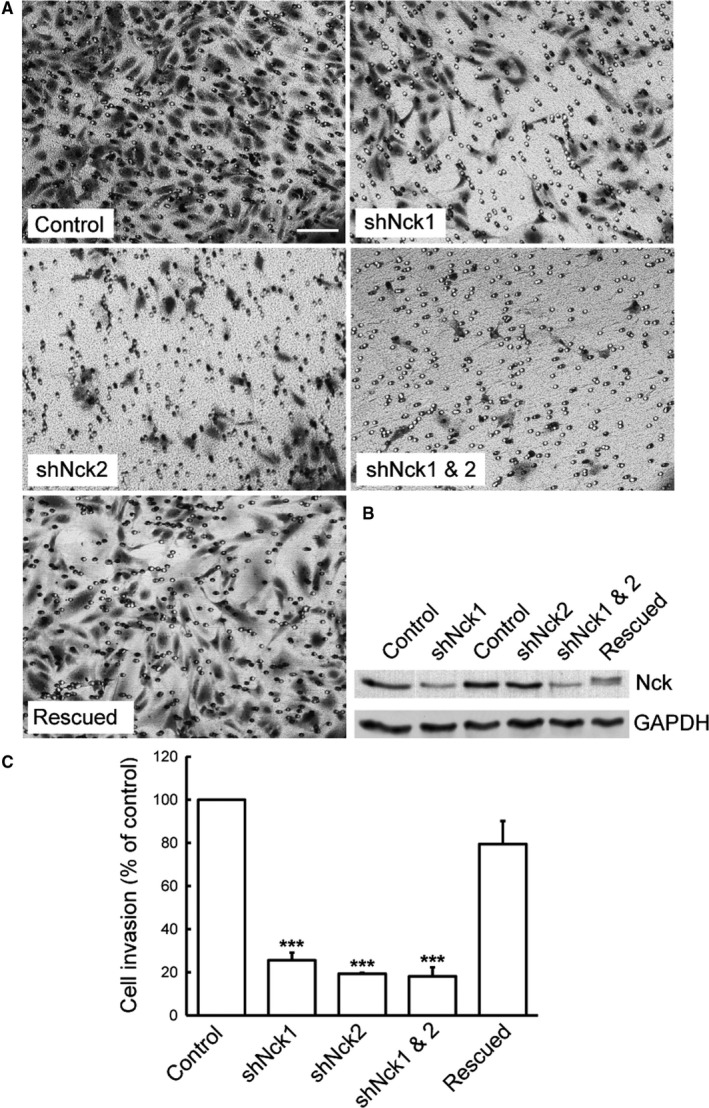
Abrogation of Nck signaling impairs endothelial cell invasion. A, Representative images of HUVEC invading through a porous membrane (8 μm pore diameter) precoated with a thin layer of Matrigel^®^ (125 μg/mL). Serum‐starved cells (4 × 10^4^ cells) were seeded and incubated for 5 h. The top and bottom compartments of Boyden chambers in 24 well plates are filled with starvation medium or starvation medium supplemented with 50 ng/mL of VEGF, respectively. After incubation, nonmigrating cells were removed, and migrating cells were fixed and stained. Brightfield images from multiple fields/treatment were collected. Scale bar, 200 µm. B, Representative western blot showing Nck and GAPDH (loading control) protein levels. C, Quantitative analysis of endothelial cell invasion. Images were processed using the EMBL ImageJ software, and cell counts were expressed as a percentage of controls. Bars represent mean ± SEM (n = 3 independent experiments). ***P < .001 vs control

Endothelial cell migration and invasion through ECM is prerequisite for angiogenic sprout formation, lumenogenesis, and vessel stabilization.^43^ As Nck adaptors are involved in podosome‐mediated ECM degradation of endothelial cells, we asked if Nck adaptors would modulate chemotactic migration and invasion of endothelial cells. Transwell invasion assays were performed using Matrigel^®^‐coated Transwell insert in the presence of a VEGF gradient. Consistent with the findings of ECM remodeling, Nck‐depleted cells exhibited a significant decrease (*P* < .001) in invasion/chemotactic migration of endothelial cells relative to control cells (Figure 4). The simultaneous depletion of Nck1 and Nck2 did not cause a further decrease in invasion/chemotactic migration when compared to single knockdown (overall 60%‐80% decrease in an invasion than control cells). Partial rescue of Nck1/Nck2 double knockdown cells with siRNA‐resistant Nck2 restored their migration/invasion capacity at levels comparable to those of control cells indicating the requirement of podosome‐mediated remodeling of ECM by Nck in chemotactic migration and invasion.

### 3.6 Cancer tissues and cells over‐express Nck

**Figure 5:**
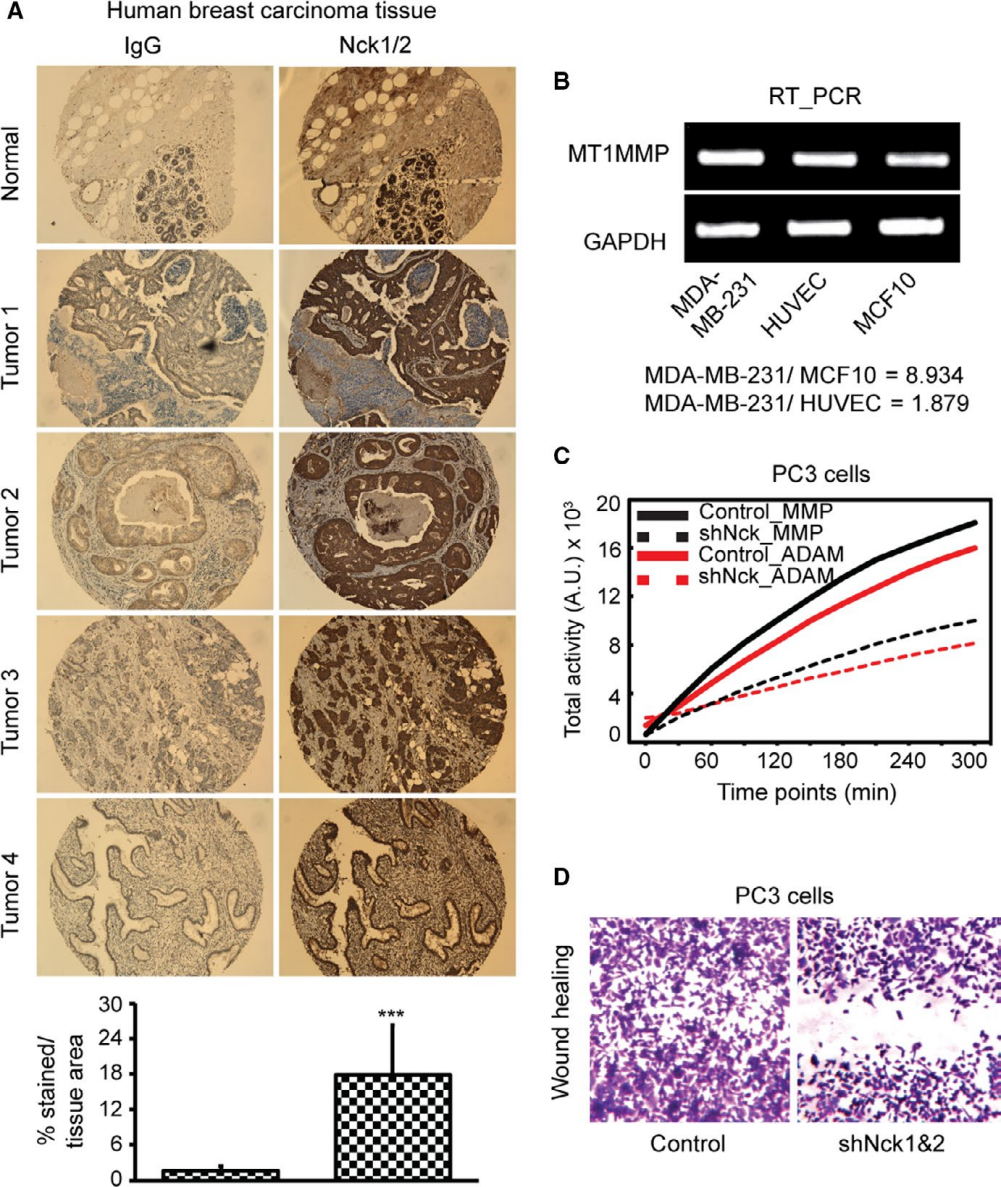
Nck expression and metalloprotease activity in cancer. A, Immunohistochemical analysis using anti‐Nck polyclonal antibody demonstrates Nck overexpression (right panel) in human ductal breast carcinoma tissues as detected by brightfield imaging of tissue micro array paraffin sections (5 μm). The left panel shows nonspecific background staining of corresponding IgG control sections.  Quantitative image analysis showing significant increase in Nck stained area (***P<.001) compared with normal tissue area (bottom panel). B, Semi‐quantitative RT_PCR of MT1‐MMP in normal and cancer cells. MT1MMP expression was higher in MDA MB‐231 cancer cells compared with HUVEC or MCF10 normal cells. RNA expression levels were normalized to the expression of the reference gene GAPDH, which did not show differential expression between the groups. C, Total MMP and ADAM activities in PC3 cells in vitro. Time‐lapse imaging of PC3 cells, incubated with MMP or ADAM specific fluorogenic substrate, demonstrate higher activities in control vs Nck‐silenced cells. D, Wound healing capacity pf PC3 cells. Nck silencing prevented PC3 cell 2D migration and wound healing. ADAM, a disintegrin and metalloprotease; MMP, matrix metalloproteinases; PC3, prostate cancer cell line; RT, reverse transcriptase

In our previous experiments, we have seen that Nck enhances the podosome formation and podosome‐mediated ECM degradation as well as invasive cell migration. An essential step in metastasis is tumor cell invasion through ECM.^44^ Although, podosomes have been demonstrated in several human cancer cell lines, particularly invasive breast carcinomas and melanomas,^45^ mechanism of their assembly is still obscure. Nck is a ubiquitously expressed adapter protein;^46^ and to determine if Nck levels alter in human cancer, we performed immune staining of Nck in human breast carcinoma tissue array sections. Both normal and metastatic tissues showed Nck protein expression. However, a higher level of Nck expression was evident in all different types of breast carcinoma tissues (Figure 5A), suggesting a role of Nck in the invasiveness of breast carcinoma. As matrix metalloproteinases (MMPs) render matrix degradation properties of podosomes, we measured membrane‐bound MT1MMP mRNA expressions and secretory MMP2 mRNA expressions in invasive cell line MDA‐MB‐231 and compared with normal cells like HUVEC and MCF10. Expression of membrane‐bound MT1MMP or MMP14 by semi‐quantitative RT‐PCR revealed higher expression of MT1MMP or MMP14 in MDA‐MB‐231 cells compared with HUVEC or MCF‐10 cells (Figure 5B). We were able to detect MMP2 (secretory) expression in HUVEC (more) and MCF10 (less) but not in MDA‐MB‐231 cell lysates (Figure 5B), suggesting that MMP expression is likely to be linked to podosome formation and the invasiveness of these cells.

### 3.7 Total MMP and ADAM activities in PC3 cells

We used FS‐6 and ADAM (a disintegrin and metalloprotease) fluorogenic substrates to measure total MMP and ADAM activities in a prostate cancer cell line (PC3) in vitro. Interestingly, we found significant (*P* < .001) decrease in both MMP and ADAM activities in Nck‐silenced group in a time‐lapse fashion when compared with control cells (Figure 5C), which was further reflected in the impairment of PC3 2D cell migration on fibronectin‐coated matrices (Figure 5D). Taken together, these data suggest that MMP and ADAM activities execute Nck‐induced podosome formation and ECM degradation.

### 3.8 Nck/Dok1 interaction is required in podosome biogenesis

**Figure 6:**
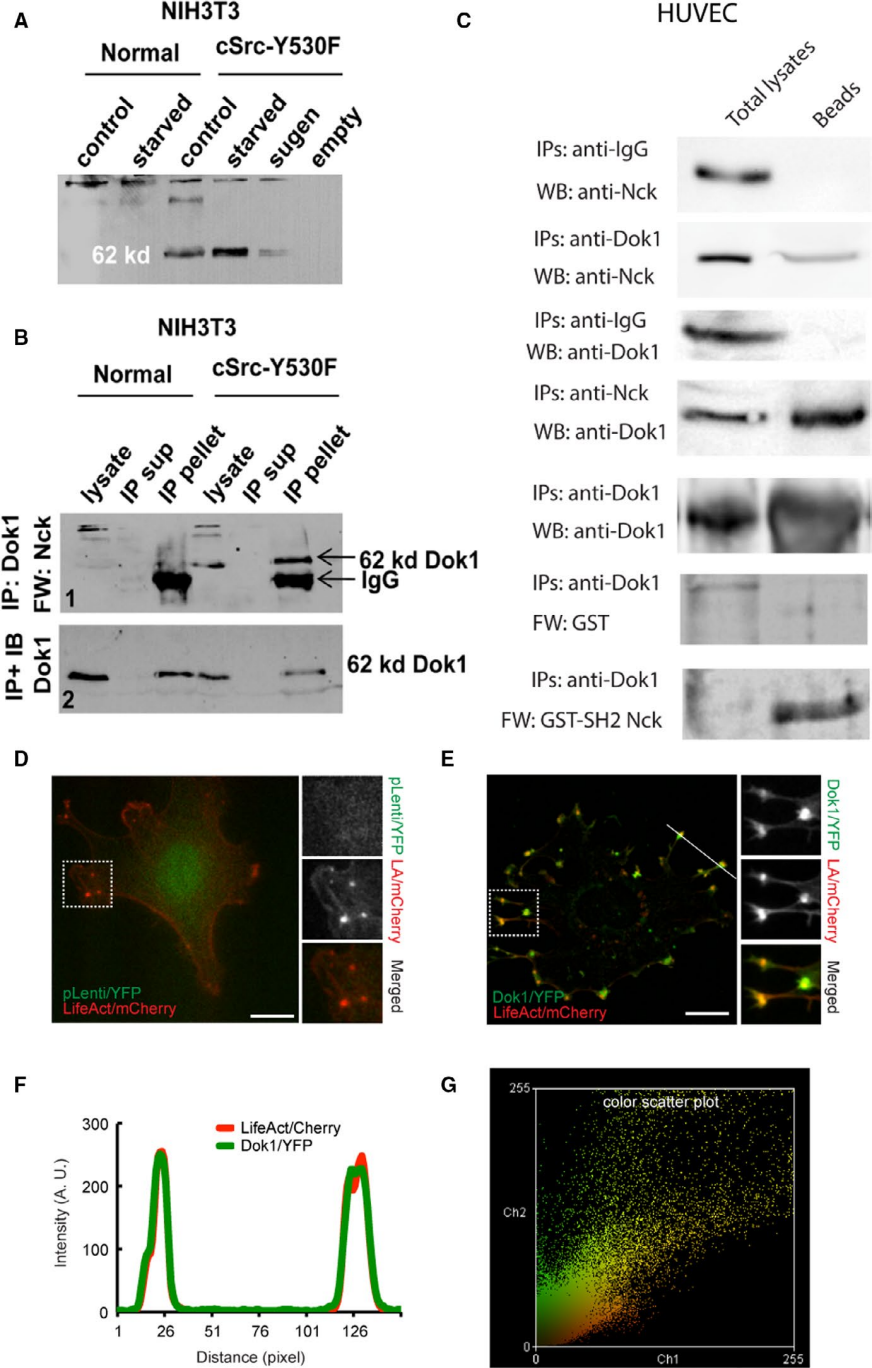
Determination of Nck and Dok1 interaction in NIH3T3 fibroblasts and Human Umbilical Vein Endothelial Cells. A, SH2 profiling of Nck by far‐western blotting in normal NIH3T3 cells and transformed 3T3 cells. A 62 kD band is consistently observed in the transformed cells forming the actin‐rich invasive structures. Although there are other bands in this blot, this particular brand is very distinctly observed in transformed cells. B, Determination of 62 kD band as p‐62 Dok. Protein analysis of normal and transformed 3T3 cells after immunoprecipitation with α‐Dok and SH2‐profiling by far‐western blotting (top); Efficiency of the IP is checked by immunoblotting with α‐Dok (bottom). C, Biochemical interaction of Nck and Dok1 in human endothelial cells. Immunoprecipitation and far‐western blotting revealed biochemical interaction between Nck and Dok1 in human endothelial cells cultured in vitro. D, Representative TIRF images of HUVEC coexpressing LifeAct/mCherry and pLenti/YFP (empty vector). Boxed area is shown on the right at higher magnification. E, Representative TIRF images of HUVEC co‐expressing LifeAct/mCherry (actin/podosomal marker) and hDok1/YFP. Boxed area is shown on the right at higher magnification. F, Line‐scan, corresponding to the white line in image (E), reveals a tight correlation of LifeAct/mCherry and hDok1/YFP fluorescence intensity at podosomes. G, Color scatter plot corresponding to the whole‐cell image shown in (D). Dok1, downstream of kinase 1

The scaffolding protein Dok1, a major substrate of Src and Abl, has been involved in cell proliferation and cytoskeletal rearrangements.^21^ Phosphorylation of Dok1 triggers Nck recruitment and filopodia formation.^22^ Also, studies showing inhibition of migration of B16F10 mouse melanoma cells^23^ by dominant‐negative mutants of Dok1 suggest a vital role of this scaffolding protein in cytoskeletal pathways controlling cells motility and invasion. Thus, it is crucial to study the formation of a Dok1/Nck complex in podosome biogenesis. In this study, far‐western blotting using Nck SH2 domain combined with immunoprecipitation identified Dok1 as an important Nck binding partner in cSrc‐transformed podosomes forming 3T3 cells (Figure 6A,B). Biochemical interaction of Dok1/Nck is also demonstrated in endothelial cells incubated in the presence of VEGF (Figure 6C).

Once we established biochemical interactions of Dok1/Nck in podosome forming cells, we analyzed Dok1/YFP localization in podosome‐forming cells. TIRF microscopy of Src‐transformed endothelial cells co‐expressing LifeAct/mCherry showed colocalization of Dok1/YFP in podosomes (Figure 6E‐G). A high correlation of colocalization is reflected in fluorescence overlay images (Figure 6E), intensity plot (Figure 6F), and scatter plots (Figure 6G), with a Pearson's correlation coefficient value of 0.68 ± 0.103 (mean ± SD, n = 11 cells). Cells co‐expressing negative control plasmid/YFP and LifeAct/mCherry did not show YFP localization in podosomal structures indicating high specificity (Figure 6D).

**Figure 7:**
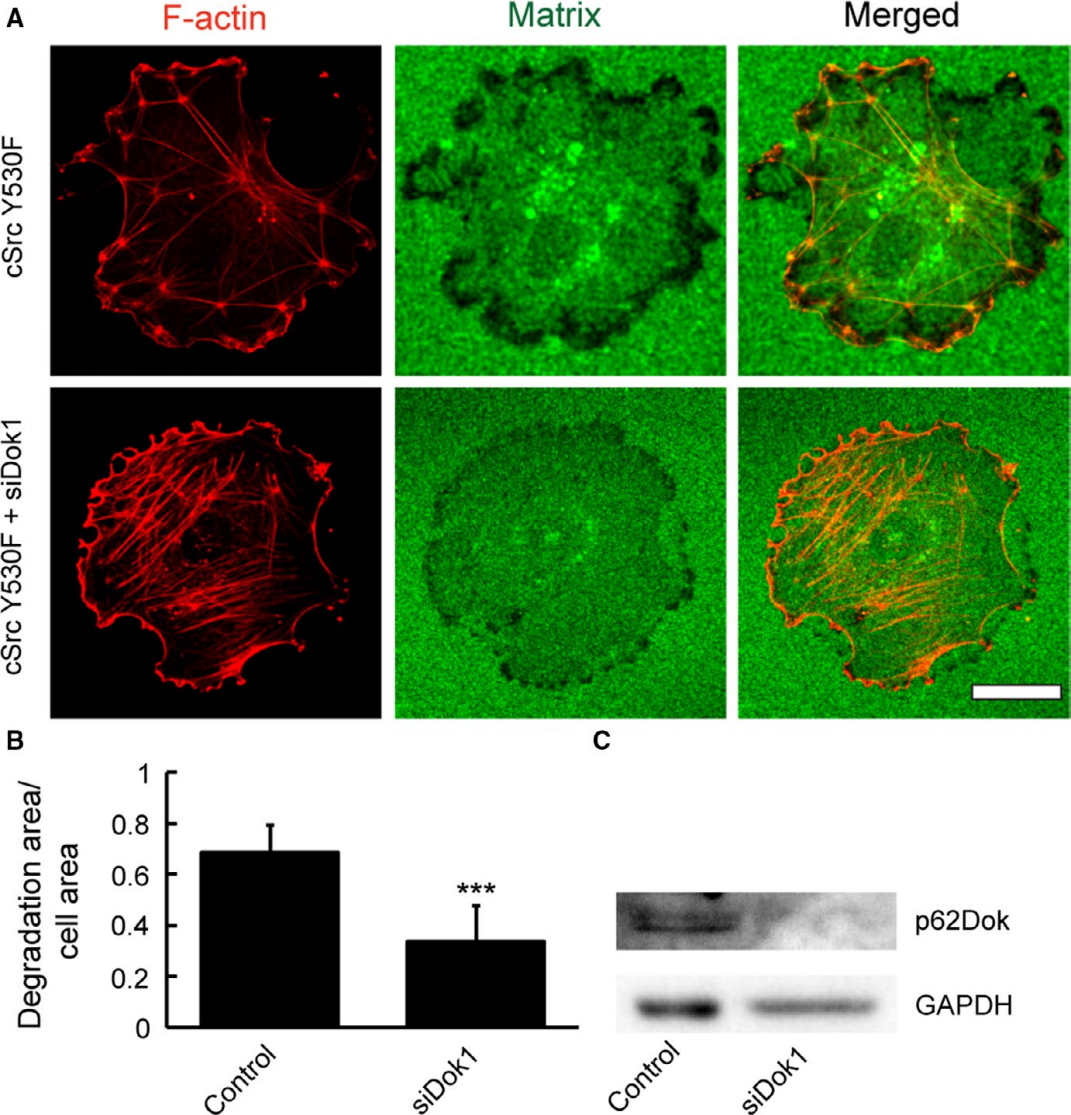
Silencing of Dok1 disrupts Src‐induced podosome formation and gelatin matrix degradation. A, Representative confocal images are showing the degradation of a fluorescent matrix (Alexa Fluor 488‐conjugated gelatin) by podosomes induced by Src expression in HUVEC. Control cell exhibit increased matrix degradation relative to Dok1‐silenced cells. Scale bar represents 20 µm. B, Quantitative image analysis showing decreased (***P < .001) matrix degradation area/cell area in Dok1‐silenced cells vs control cells. Bars represent mean ± SD (n = 3 independent experiments). C, Western blot of control and Dok1‐silenced endothelial lysates. Dok1, downstream of kinase 1

In order to assess the effect of Dok1 silencing on podosome‐mediated ECM degradation, cSrcY530F‐transformed endothelial cells were treated with siDok1and subjected to fluorescent gelatin matrix degradation. Confocal images show inhibition of matrix degradation by Dok1 silencing endothelial cells (Figure 7A). A significant (*P* < .001) decrease in fluorescent gelatin matrix degradation area by Dok1‐silenced Src‐transformed endothelial cells were observed when compared with control Src‐transformed endothelial cells (Figure 7B). Western blot analysis indicates >90% silencing of Dok1 in cSrc Y530F‐transformed endothelial cells (Figure 7C). Similarly, Dok1 silencing in 3T3 fibroblasts, through the retroviral expression of shDok1, prevented podosome formation and matrix degradation (Figure S4). Collectively, our results suggest an important functional role of Nck/Dok1 interaction in podosome biogenesis.

## 4 DISCUSSION

Actin‐rich podosomes can degrade ECM required in invasive cell migration.^47^ Podosomes contribute to cell migration, chemotaxis, transmigration, migration through vascular basement membrane,^48^ tumor angiogenesis,^10^ cellular immune response of macrophage migration toward infectious agents to clear pathogens,^49^ as well as pathological processes like cancer metastasis. Podosomes formation requires when monocytes traffic from the blood, macrophage, and dendritic cell migration through tissues.^50^ Podosomes are also required in endothelial transmigration, invasion, and chemotaxis.^51^ Although podosome formation is important in many different physiological processes, the molecular mechanism of podosome biogenesis is still poorly understood. Several molecules and signaling pathways are involved in the process and understanding all these molecular machinery is difficult but necessary for developing new therapeutic agents to treat life‐threatening diseases like cancer and cardiovascular disorders. Previous literature suggests that actin remodeling of Nck is involved in T‐cell receptor activation,^52^ invadopodia formation in tumor cells,^15^, ^18^, ^40^ Fas ligand transport to synapse,^53^ and the organization of phagocytic cups.^54^ Nck1 and Nck2 are –SH2 and –SH3 domain‐containing adaptor proteins that we have previously shown to be involved in remodeling of actin cytoskeletal dynamics to promote directional cell migration^30^ and endothelial lumen formation.^25^ Actin nucleation and branching is initiated by the Arp2/3 complex.^55^, ^56^ Arp2/3 inhibitor CK666 inhibited the podosome formation in monocytes and megakaryocytes.^55^, ^57^ In living cells, clustering of Nck stimulated the Arp2/3‐mediated actin polymerization through the activation of WIP/N‐WASp.^58^, ^59^ Applying molecular genetics and fluorescence microscopy, here we demonstrate that Nck adaptor proteins colocalize and induce the podosomes biogenesis in endothelial cells in the presence or absence of Src transformation. Our data show that ectopic expression of Nck induces the formation of podosomes in endothelial cells that do not normally have them without any stimulation in vitro, suggesting that Nck is required in podosomes formation. Time‐lapse live‐cell imaging revealed that Nck‐induced podosome formation follows de novo synthesis, fission, and fusion, supporting the characteristics observed in Src‐transformed fibroblasts, as demonstrated recently.^35^ Recently, it was demonstrated that vascular endothelial cells form larger podosomes on more rigid substrates.^6^ Signals that induce podosomes, such as integrin engagement or phorbol ester treatment, act mainly via triggering Src activation. Constitutively active Src expression directly induces podosome formation,^60^ while depletion of Src in osteoclasts prevented podosome belt formation.^61^ Surprisingly, in our present study, Src‐transformed endothelial cells lacking Nck were unable to promote the podosome formation and podosome‐mediated fluorescent gelatin matrix degradation. Furthermore, ectopic expression of Nck in Src‐transformed endothelial cells enhanced ECM degradation, suggesting an essential role of Nck in Src‐induced podosome formation. ECM degradation is prerequisite for invasive cell migration for normal physiological processes like angiogenesis or under pathological conditions like cancer metastasis. In our study, using a collagen‐coated transwell membrane, we demonstrated VEGF induced the invasive cell migration of endothelial cells under endogenous levels of Nck. However, endothelial cells lacking Nck1 or Nck2 or both showed significant loss of invasive cell migration capability, which was restored by ectopic expression of mNck2. Invasion through ECM is prerequisite in malignancy to form secondary growth.^62^ Podosomes, capable of invasive cell migration, have been demonstrated in several human cancer cell lines, particularly in invasive breast carcinomas and melanomas, and their presence has been correlated with invasiveness in vitro.^63^, ^64^, ^65^ The –SH3 domain‐containing adaptor protein Tks5/Fish has been shown in podosome and invadopodia formation^66^, ^67^ (as well as cancer cell invasion).^68^ Metastasis or cancer cell invasion to adjacent tissues is the leading cause of mortality in cancer patients.^69^ In our present study, once we established that Nck regulates podosome formation and invasive cell migration, we analyzed Nck levels in human breast carcinoma tissues. The immunohistochemical analysis revealed very high levels of Nck expression in different types of human breast carcinoma tissues. MMPs are key players in ECM remodeling during cancer metastasis.^70^ Podosomes are rich in MMPs that drive matrix remodeling in various cell types.^71^ In a recent study, using human breast carcinoma cell line MDA‐MB‐231 and mouse model, we demonstrated that Nck facilitates MMP14‐mediated breast carcinoma cell invasion.^12^ In our present study, RT_PCR analysis revealed high MMP14 expression in MDA‐MB‐231 cells compared with noncancerous cells MCF‐10 and HUVEC (Figure 5B). Other metalloproteases like ADAMs play roles in mediating cell‐cell and cell‐matrix interactions in both normal development and pathological states such as Alzheimer's disease, arthritis, cancer, and cardiac hypertrophy.^72^ The adaptor protein Fish have been implicated in association with members of the ADAMs family and localizes to podosomes of Src‐transformed cells.^66^ When we performed in vitro analysis of MMP14 and ADAM10 activities in a PC3, we observed higher levels of activities under endogenous levels of Nck compared with Nck‐silenced cells (Figure 5C) that further reflected in wound repair capacity in 2D fibronectin matrices (Figure 5D).

Once we analyzed podosomal function with respect to ECM remodeling as well as invasive cell migration and metalloprotease activities under different levels of Nck, we identified the molecular interaction of Nck with Dok1 in podosome formation. Dok1 is a 62‐kDa protein that is phosphorylated by both receptor and nonreceptor tyrosine kinases.^73^, ^74^ Dok1 upregulated in the invasive cell population.^75^ Also, in colorectal cancer, the nuclear localization of Dok1 correlated with poor outcome.^76^ Although Nck has been reported to bind to Dok1,^77^ the role of Nck/Dok‐1 interaction in podosome formation has not yet been explored. Indeed, our co‐immunoprecipitation experiment and far‐western blotting clearly show an association between Nck and Dok1 in podosome‐forming cells. Nevertheless, we show herein that Dok1 localizes to podosomes and is required for podosome formation. Dok1 silencing significantly affected podosome formation and ECM degradation in both endothelial cells and fibroblasts (Figure 7 and Figure S4).

Overall, our study highlights a novel role of Nck in regulating podosome biogenesis. We have shown that Nck is required for podosome biogenesis in normal, as well as cSrcY530F‐transformed cells. Nck gets overexpressed in breast cancer tissues, and cancer cell lines enhancing MMP‐mediated ECM remodeling. Biochemical interaction of Nck/Dok1 in podosome forming cells has been identified.

## 5 CONCLUSION

This study discovered a new role of Nck in podosome biogenesis that therefore be used as an in vitro model of enhanced podosome formation in cells that do not usually have them. Silencing of Nck can block podosome‐mediated ECM degradation preventing cancer invasion and metastasis. Importantly, Nck overexpression in tissue biopsies can be used as a biomarker of metastatic cancer.

## CONFLICT OF INTEREST

Sankar P. Chaki, Rola Barhoumi, and Gonzalo M. Rivera declare that they have no conflict of interest.

## AUTHOR CONTRIBUTIONS

Sankar P. Chaki conceived, designed, and performed experiments, and wrote the manuscript. Gonzalo M. Rivera conceived and designed the experiments, and provided reagents. Rola Barhoumi assisted in imaging experiments.
