# Supplementary figures and images for: Nck adapter proteins promote podosome biogenesis facilitating extracellular matrix degradation and cancer invasion
Source: Cancer Med. 2019 Oct 22;8(17):7385–98. doi: 10.1002/cam4.2640 (PMC6885876; doi:10.1002/cam4.2640)

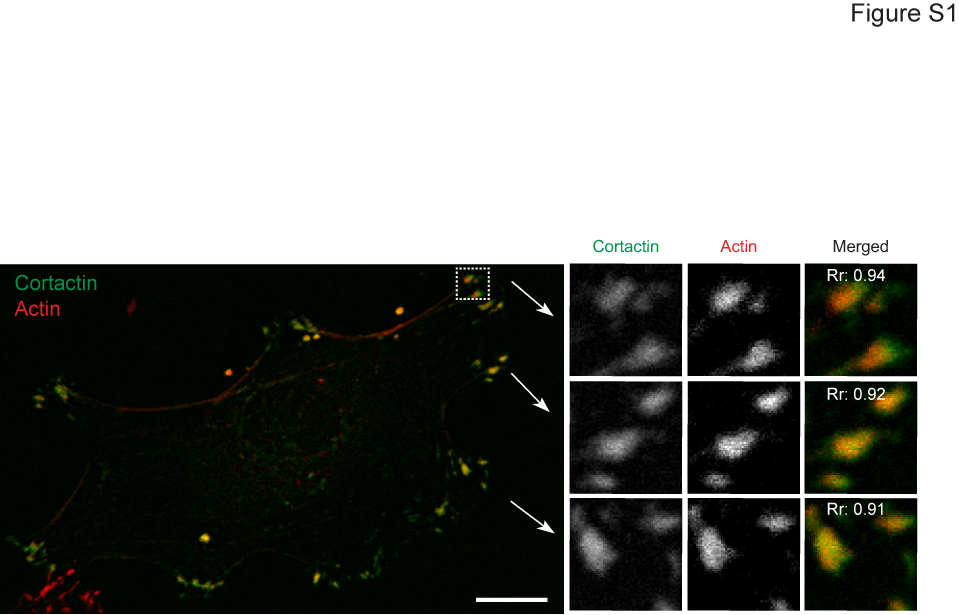

Supplement: Supplementary file 1 [file CAM4-8-7385-s001.tif]

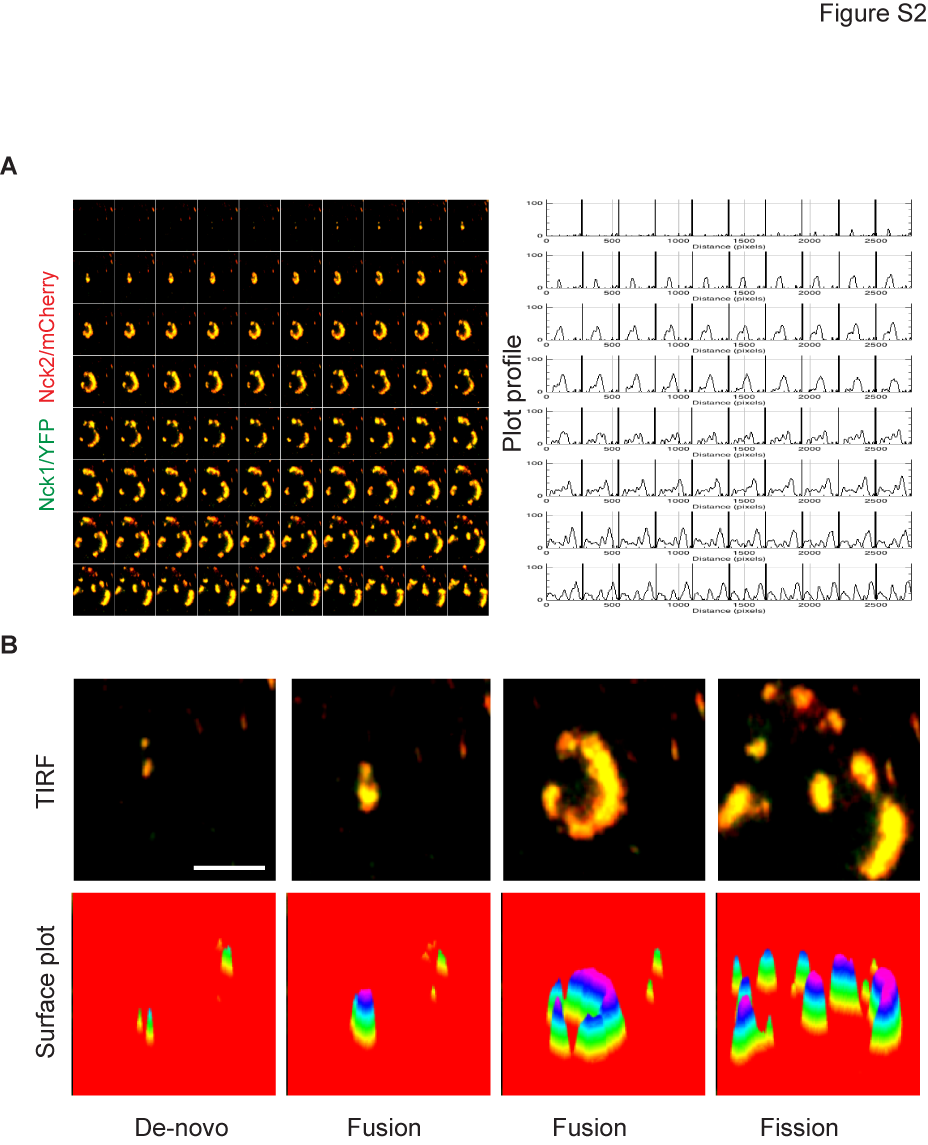

Supplement: Supplementary file 2 [file CAM4-8-7385-s002.tif]

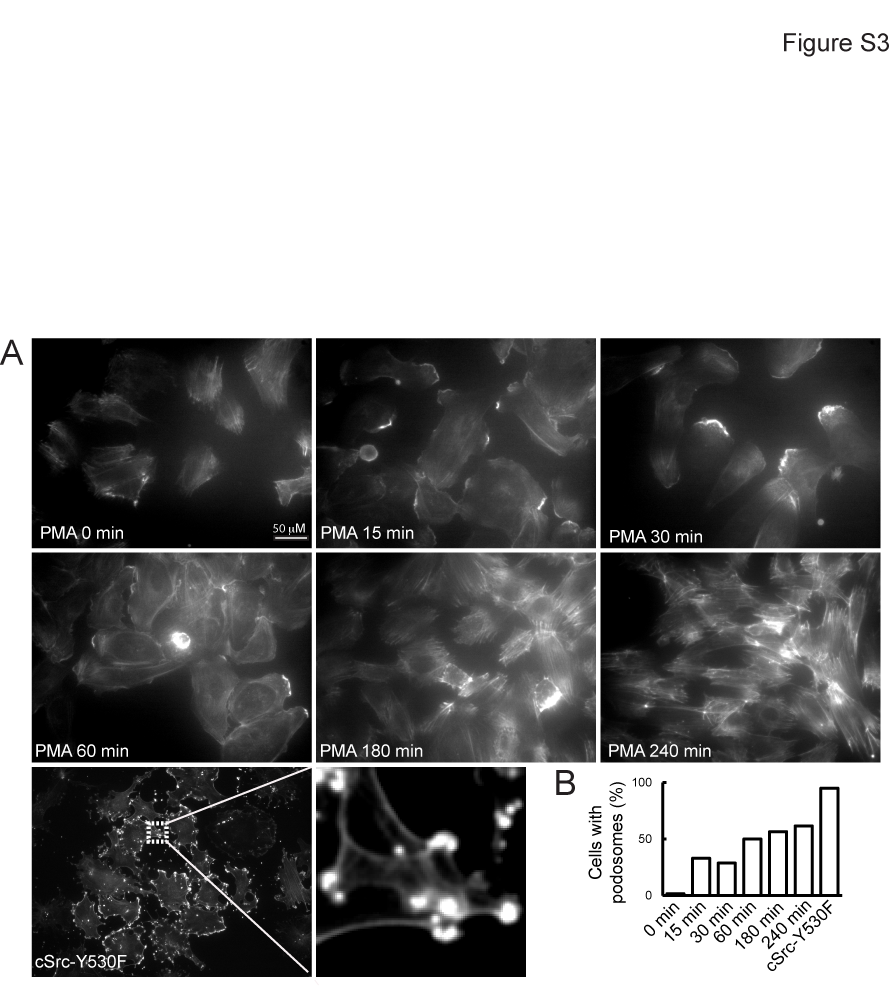

Supplement: Supplementary file 3 [file CAM4-8-7385-s003.tif]

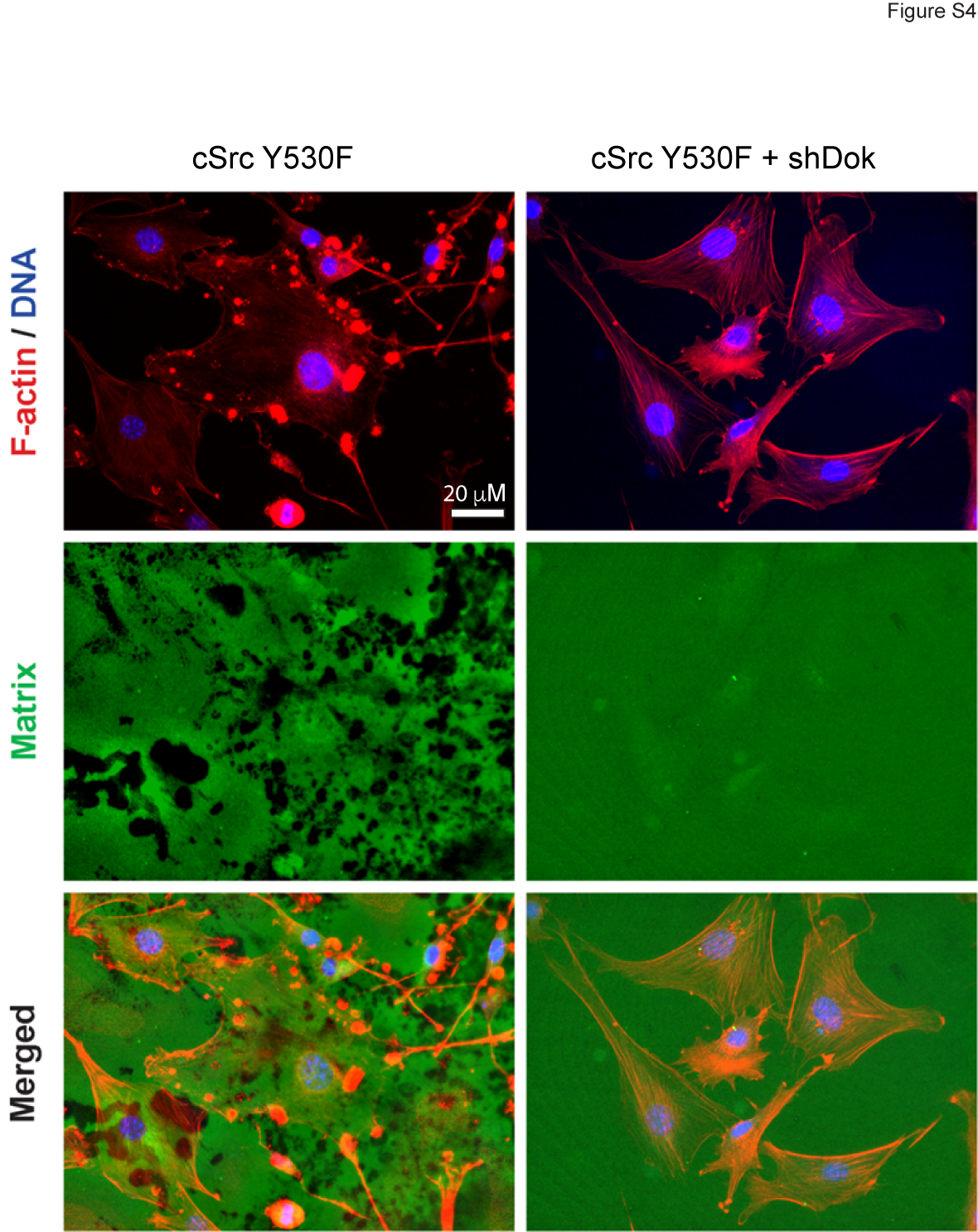

Supplement: Supplementary file 4 [file CAM4-8-7385-s004.tif]
